# Promoting self‐efficacy in patients with chronic disease beyond traditional education: A literature review

**DOI:** 10.1002/nop2.382

**Published:** 2019-10-20

**Authors:** Holly Farley

**Affiliations:** ^1^ Eastern Illinois University Charleston IL USA

**Keywords:** education, long‐term illness, nurses, nursing, self‐efficacy

## Abstract

**Aim:**

To examine barriers to self‐efficacy and strategies beyond traditional education that promote self‐efficacy for patients living with chronic disease. The review questions were as follows: (a) What are barriers to self‐efficacy in patients experiencing chronic disease? and (b) What non‐traditional strategies and programmes can be implemented by healthcare leaders to promote self‐efficacy in patients with chronic disease?

**Design:**

Integrative review.

**Method:**

Data sources searched were CINAHL, Google Scholar, Health Source, Academic Search Complete and PsycARTICLES published between January 2014–January 2018. Synthesis and thematic analyses were conducted on 24 articles.

**Results:**

Three themes were identified as barriers to self‐efficacy: health literacy, access and support. Four prominent strategies were found to promote self‐efficacy: self‐management programmes, telehealth, mobile applications, gaming and social media. The findings indicate self‐efficacy for patients with chronic conditions can improve with new interventions. Enhancing traditional education and boosting self‐efficacy could increase treatment adherence and decrease cost.

## 1 INTRODUCTION

In the USA, six in ten adults were diagnosed with a chronic disease. Furthermore, it was determined that four in ten adults have two or more chronic diseases (“About chronic diseases/CDC,” 2019). A wide range of physical and psychological symptoms as well as significant lifestyle changes can affect chronic disease patients. Further, chronic diseases cause a health and healthcare expenditure burden in the United States: according to the Center for Disease Control and Prevention, 90% of the nation's 3.3 trillion dollars in annual healthcare expenditures are reserved for people with chronic and mental health conditions (2019). Increasing lifespans and the shift from physician‐managed care to patient‐managed care has created the need to promote self‐management of disease processes and improve outcomes (Cameron et al., 2018). In addition, patients are expected to take a more active role in their medical care and be knowledgeable about and manage diseases (Anekwe & Rahkovsky, 2018; Cameron et al., 2018; Henselmans, Heijmans, Rademakers, & Van Dulman, 2014; van Berkel, Lambooij, & Hegger, 2015).

Self‐efficacy is described as a cognitive process where, through environmental influence and social influence, individuals learn new behaviours that affect their ability to improve future events (Bandura, 1977). Promoting self‐efficacy can improve the outcomes and quality of life for patients living with chronic diseases (Wu, Hsieh, Lin, & Tsai, 2016). Traditional forms of education that include a discussion between the patient and caregiver, as it is well documented in the literature, are not sufficient in providing patients with the necessary understanding and skills to manage their disease and minimize complications (van Berkel et al., 2015). This literature review aims to identify the barriers to self‐efficacy and promote self‐efficacy by exploring non‐traditional strategies that can be implemented in healthcare settings.

### 1.1 Background

Managing chronic diseases in an aging population is complex (Anekwe & Rahkovsky, 2018) and require various strategies (Cameron et al., 2018). The many recent changes in health care—increased access to care and treatment options, evidence‐based practice, the shift to considering patient preferences and expecting patient autonomy in care decisions—have expanded the role of the patient. Patients are now supposed to be the driver of their health care and adhere to regimens with the hope of maintaining health and decreasing complications (Bratzke, 2015; Koch, Wakefield, & Wakefield, 2015; Win, Hassan, Oinas‐Kukkonen, & Probst, 2016). Further, Bratzke (2015) found that a patient diagnosed with one chronic disease can make as many as 20 choices related to a single disease. Since clients manage many chronic diseases and regimens and collaborate with multiple specialty providers, day‐to‐day management can prove to be extremely complex.

Bandura (1977) describes self‐efficacy as an efficacy expectation where a person believes in taking a particular action and producing a specific outcome. Self‐efficacy is not a trait, but rather a set of beliefs. People must believe they can produce certain effects with their actions or they will not persevere in difficult situations. People's beliefs in their efficacy have a direct impact on meeting one's goals (Bandura, 2018). Therefore, to improve self‐management of disease processes, patients must have increased self‐efficacy and believe they can manage their disease (s) (Bandura, 2005; Cutler, Crawford, & Engleking, 2018; van Berkel et al., 2015; Wilson et al., 2018). Furthermore, Anekwe and Rahkovsky (2018) argue that any patient with a chronic disease, no matter the type, needs a skill set for managing it and self‐efficacy is one of them. Self‐efficacy is a mediator between knowledge and self‐care (Wu et al., 2016), and exploring strategies to boost self‐efficacy will improve health outcomes in patients navigating chronic diseases (Cutler et al., 2018; Fors et al., 2018; Willis, 2016).

Traditionally, patients receive information from their primary care physician via verbal or written communication during an office visit. These are considered passive approaches to education and, although they may facilitate increased understanding, they do not ensure increased self‐efficacy or behaviour changes (Devan, Hale, Hempel, Saipe, & Perry, 2018). Some common barriers to this form of education include time constraints, availability of knowledgeable staff, health literacy and patient's understanding and readiness to learn (Henselmans et al., 2014; Rivera, 2017). Wu et al. (2016) argue against simply stopping at clinical education. Rather, the integration of active self‐management and interventions that improve self‐efficacy should be addressed, beginning with acute care, primary care and care coordination. This takes the careful assessment and the implementation of interventions beyond face‐to‐face education.

### 1.2 Research questions

The following questions were used to guide the review: (a) What are barriers to self‐efficacy in patients experiencing chronic disease? and (b) What non‐traditional strategies and programmes can be implemented by healthcare leaders to promote self‐efficacy in patients with chronic disease?

### 1.3 Design

An integrative literature review was used and allows for the potential to build nursing science and inform initiatives including research, practice and policy (Wittemore & Knafl, 2005). Experimental and non‐experimental studies were used to better answer the research questions. This approach allows the most comprehensive data collection to analyse the phenomenon thoroughly (Souza, Silva, & Carvalho, 2010).

### 1.4 Method

For this review, chronic disease was defined as a disease process that cannot be cured. More specifically, the Center for Disease Control (CDC) identifies several diseases as chronic diseases, broadly defining their conditions as those that last 1 year or more, require ongoing medical attention or limiting activities of daily living or both ("About chronic diseases/CDC,” 2019).

**Figure 1:**
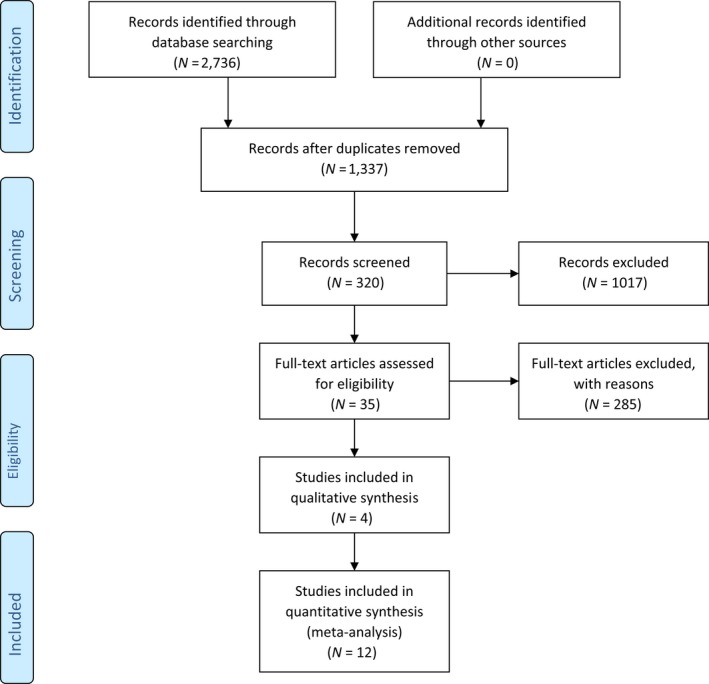
Literature search selection diagram

The search was conducted using the following electronic databases: Nursing and Allied Health Literature (CINAHL), Google Scholar, Health Source: Nursing Academic Edition, Academic Search Complete and PsycARTICLES. Initial search of key terms *Self‐efficacy and Chronic Disease* yielded articles that used other and/or additional terms to describe self‐efficacy and specific interventions. This prompted a deeper search with terms ‘self‐management’, ‘empowerment’, ‘self‐care’, ‘telehealth’, ‘social media’ and ‘mobile health’. Boolean operators were used to maximize results of the search and the time frame for the review was limited to 5 years, January 2014–January 2018 (Figure 1). The studies included were limited to peer‐reviewed publications in English that focus on adults aged 18 and above and on the promotion of self‐efficacy in patients with one or more long‐term illnesses. One study included in the review used participants equal or greater than 15 years of age. This study was included due to the large number of participants (*N* = 1,314) and mean age being 63.4 years (Henselmans et al., 2014).

All literature was mined for information that could be generalized to anyone with a chronic disease to identify the barriers and the most successful strategies to improve self‐efficacy and patient outcomes. Furthermore, selected literature reviews were assessed for duplication of studies used and none were identified (Table 1).

**Table 1:** Summary of selected studies included in the review

Author/Year	Design	Purpose/Aim	Setting/Sample	Intervention	Results and findings
Baron and Newman (2016) United Kingdom	Randomized Controlled Trial	Investigated behavioural effects of mobile phone‐based telehealth	Adults (*N* = 81) with poorly controlled type 1 and type 2 diabetes	Compared mobile phone application and standard care	Mobile phone application improved the management of diabetes and self‐care. Significantly improved self‐efficacy
Bratzke (2015)	Narrative Review	Synthesize research findings related to self‐management	Thirteen articles Majority qualitative Participants with multimorbidity	Empirical studies addressed priority setting and decision‐making	Decisions based on personal preferences and values, barriers are prevalent and focused research needed
Cameron et al. (2018) Canada	Quantitative correlational	Evaluate the effectiveness of telehealth in remote patients with chronic conditions	Adults (*N* = 213) Rural and remote with chronic disease	Chronic disease self‐management programme via teleconference	Patients need content delivered based on social identification provides for increased self‐efficacy
Cottrell et al. (2017)	Systematic review and meta‐analysis	Evaluate the effectiveness of real‐time telerehabilitation	13 studies met the criteria for methodological criteria	Adults with a diagnosed musculoskeletal condition. Treatment intervention via real‐time telerehabilitation medium compared with face‐to‐face	Telerehabilitation is effective in improving physical function, disability, and pain
Cutler, Crawford, & Engleking (2018)	Systematic review	Evaluate outcomes of self‐management for adults with chronic conditions	Ten articles investigating the management of chronic conditions	Self‐management programmes provided through in‐person, group sessions	Improving self‐efficacy through self‐management can have a direct or indirect impact on behaviour change. Behaviours must be sustained to sustain change
Devan et al. (2018)	Systematic review and meta‐synthesis	Synthesize enablers and barriers to self‐management	33 studies (*N* = 512) Thematic analysis and confidence in evidence from reviews of qualitative research approach Patients with chronic pain	Only included interventions that involved at least four self‐management skills for chronic pain	Providing intermittent support, peer support groups shared decision‐making and guided problem‐solving is essential to ongoing self‐management
Fors et al. (2018) Sweden	Quantitative Descriptive	Evaluate the effects of person‐centred support via telephone	Adults (*N* = 221) with COPD and/or CHF measured general self‐efficacy, re‐hospitalization, and death	The intervention was person‐centred telephone support from educated RN with specific training after discharge. Control group received no additional care	Intervention reduced risk of decreased self‐efficacy and clinical events were not increased up to 6 months post‐discharge
Fortin et al. (2019) Canada	Descriptive Qualitative	Evaluate the integration of chronic disease prevention and management services into primary care practices. Corroborate quantitative results	Interviews with patients (*N* = 36), family members (*N* = 2), Focus groups (*N* = 7), healthcare professionals (*N* = 16) multimorbidity	Chronic disease prevention and management programme by an interdisciplinary team integrated into existing self‐management programme	Increased awareness, knowledge, and increased motivation and empowerment. Positive impact on patients and family members. Negative effects were the loss of beneficial effects of intervention and support or resistance from family modulated effects
Hardinge et al. (2015) United Kingdom	Quantitative Descriptive	Evaluate the effectiveness of a mobile telehealth application that allows patients to record data, communicate with healthcare professionals, and access educational materials	Participants in the mHealth intervention. Adults (*N* = 18) with moderate to severe COPD	COPD patients use mobile telehealth (mHealth) applications to monitor symptoms and use of education for the self‐initiated treatment of exacerbation	Daily use of mHealth platform was feasible and acceptable to users and was found to potentially identify exacerbations early
Henselmans et al. (2014) Netherlands	Quantitative Correlational	Examine perceived efficacy and barriers in consultations and communication support	Diagnosed chronic disease 15 years or older (*N* = 1,314)	Completed the perceived efficacy in patient‐provider interaction scale, and questions related to barriers to participation and interest in communication support	Most felt efficacious during consultations, common barriers were ‘too little time’ and concern of being bothersome.’ Patients perceived the least barriers when seeing a nurse
Hickman et al. (2015) United States	Nonblinded randomized controlled trial	To report preliminary efficacy of a serious game for health (eSMART‐HD) to enhance blood pressure control among community‐dwelling adults with hypertension	Nonprobability sample of adults with hypertension (*N* = 116)	Participants assigned to the experimental group were exposed to screen‐based education focused on hypertension and self‐management strategies	Results confirmed the efficacy of eSMART‐HD as a strategy for hypertension self‐management and improving blood pressure control
Horrell et al. (2017) United States District of Columbia Puerto Rico	Quantitative	To compare enrolment and completion rates of middle‐aged Chronic Disease Self‐management Programme with different income levels and sociodemographics	Age 55–64 (*N* = 19,365)	Chronic Disease Self‐management Programme	Results found middle‐aged participants from the most impoverished counties were more likely to complete the programme
Kennedy et al. (2017) Canada	Quantitative Statistics	Compare two modes of delivery of a prescription for an education programme with arthritis patients. In‐person and remote	Adults with arthritis (*N* = 123) participated 36 were in‐person, and 87 were remote	One‐day telemedicine programme was adapted for two video conference workshops	Similar improvements in self‐efficacy and other outcomes for both groups
Koch, Wakefield, & Wakefield (2015)	Systematic Literature Review (12 qualitative studies)	Locate and describe patient perceptions of barriers and facilitators to managing multiple chronic conditions	13 peer‐reviewed articles that evaluated the burden of care in adults with two or more chronic diseases	N/A	13 barriers and nine facilitators were found
Lin et al. (2017)	Meta‐analysis of randomized controlled studies	Evaluated the effects of self‐management programmes in patients with chronic kidney diseases	Eighteen randomized controlled trials	Self‐management programme	Small effect on self‐management, medium effect on self‐efficacy, depression, and quality of life, large effect on anxiety
Mackey et al. (2016)	Literature Review	Assess the association between health literacy and patient characteristics related to self‐management behaviours	Studies must use a valid health literacy tool, at least one self‐management behaviour assessed, and patients had a chronic condition (*N* = 31)	N/A	Low health literacy may affect behaviours, such as self‐efficacy, that are necessary for the development of self‐management skills
Roncoroni et al. (2019) United States	Quantitative Correlational	Understand the role of health self‐efficacy as a precursor to health‐promoting behaviours and treatment adherence	Rural adults (*N* = 273) Predominately low‐income and indigent rural patients	Completed demographic data questionnaire, self‐rated abilities for health practices scale, general adherence measure, and health‐promoting lifestyle profile II	Health self‐efficacy is key to adjust behaviours and improve adherence. Also, behaviours in urban patients can be generalized to rural patients
Stellefson et al. (2017) United States	Quantitative	Understand patients eHealth literacy or ability to seek, find, understand, appraise online health information and apply knowledge	Patients registered with the COPD Foundation's National Registry (*N* = 1,270)	Completed surveys on sociodemographic status, socio‐cognitive status, health status, and eHealth literacy scale	Greater disease severity is associated with higher eHealth literacy, and greater educational attainment and higher COPD‐related knowledge predicts higher eHealth literacy
van Berkel et al. (2015) Netherlands	Qualitative	Investigate whether discussions about medicine use taking place in online message boards contribute to patient empowerment and result in more effective use of medicine	Thematic deductive analysis of 5,532 posts on seven message boards related to ADHD, ALS, and Diabetes	Message boards	Found patient empowerment processes in posts for all three disorders. Type of information shared can contribute to patient self‐efficacy with medicine use
Willis et al. (2016) United States	Qualitative Ethnography	Identify factors of self‐efficacy related to self‐management behaviours found in computer‐mediated communication by people with arthritis	Sampled posts on message boards (*N* = 5,762)	Message boards	Three themes: Sharing experiences, suffering from symptoms, asking for help. Supports online health communities could facilitate self‐efficacy to practice arthritis self‐management
Wilson et al. (2018) United States	Quantitative	Evaluate participant engagement and effects of an Internet‐based, self‐direct programme for depressive symptoms among adults with chronic disease	Adults (*N* = 47)	Randomly assigned to Think Clearly About Depression online self‐management programme or control group. Completed Patient Health Questionnaire and Chronic Disease Self‐efficacy Scales	Engagement and satisfaction was favourable, depressive symptoms and self‐efficacy in the management of depressive symptoms improved for the treatment group, but not for the control group
Win et al. (2016) Australia	Quantitative	To identify patients' preference about the design features of effective online patient education and the benefits	Chronic disease patients or family members and health professionals (*N* = 215)	Online Patient Education sites (OPE)	Confirmed a set of design features to be included in OPE sites, and validated a set of health and social benefits of OPE sites
Wu et al. (2016) Taiwan	Quantitative Cross‐sectional Correlational	Investigate important factors in self‐care of chronic kidney disease (CDK) and mediating effects of self‐efficacy on knowledge and self‐care	Chronic kidney disease patients (*N* = 247)	Participants completed the CDK self‐care Knowledge Scale, CDK Self‐efficacy Scale, CDK self‐care Scale and demographic and disease characteristics survey	Self‐efficacy crucial mediator between knowledge and self‐care and healthcare professionals should offer strategies to increase self‐efficacy

Data analysis followed a methodical approach. The literature was organized to allow for comparison of primary sources and identify specific variables and characteristics related to the questions being asked. Following the literature search stage, articles were analysed, coded and summarized. Prominent codes were grouped and further reduced into themes. Organizing the data in this way resulted in the ability to identify patterns and relationships and draw conclusions (Wittemore & Knafl, 2005).

### 1.5 Ethics

As this is a review of previous literature, it was not required to receive permission from an institutional review board or ethics committee.

## 2 RESULTS

### 2.1 General description of the studies

The literature reviewed includes 24 studies including qualitative (*N* = 3), quantitative (*N* = 13), mixed methods (*N* = 1) and literature reviews (*N* = 7). Studies focused on both patients with a specific disease process, as well as, chronic disease in a general sense. Most studies inclusion criteria included the diagnosis of one or more chronic diseases. Ten articles focused on participants with a single disease: arthritis (*N* = 2), chronic obstructive pulmonary disease (COPD) (*N* = 2), chronic kidney disease (CKD) (*N* = 2), hypertension (*N* = 1), diabetes (*N* = 1), musculoskeletal (*N* = 1) and chronic pain (*N* = 1). Ten articles focused on multimorbidity, and four did not distinguish. Most of the included research studies were conducted in the United States (*N* = 6) followed by Canada (*N* = 3), United Kingdom (*N* = 2), Netherlands (*N* = 2), Australia (*N* = 1), Sweden (*N* = 1) and Taiwan (*N* = 1).

### 2.2 Interventions

The main characteristics of the sources reviewed were the implementation of an intervention to promote self‐management of the patients' disease(s) and identify behaviour effects and promotion of regimen adherence. Interventions included mobile phone applications (Baron, Hirani, & Newman, 2016; Hardinge et al., 2015), gaming (Hickman, Clochesy, Pinto, Burant, & Pignatiello, 2015), telehealth that included telephone or video streaming (Cameron et al., 2018; CottrellGalea, O'Leary, Hill, & Russell, 2017; Fors et al., 2018; Kennedy et al., 2017), self‐management programmes (Bratzke, 2015; Cutler et al., 2018; Devan et al., 2018; Fortin, Chouinard, Diallo, & Bouhali, 2019; Horrell et al., 2017; Lin, Liu, Hsu, & Tsai, 2017; Win et al., 2016), social media (van Berkel et al., 2015; Wilson et al., 2018), surveys addressing self‐efficacy (Henselmans et al., 2014; Koch et al., 2015; Roncoroni, Tucker, Wall, Wippold, & Ratchford, 2019; Wu et al., 2016) and health literacy survey (Mackey, Doody, Werner, & Fullen, 2016; Stellefson et al., 2017).

### 2.3 Findings

Research question one sought to identify barriers to self‐efficacy patients with chronic disease experience. Three main themes emerged as barriers to self‐efficacy: (a) health literacy, (b) access and (c) support. “The Patient Protection and Affordable Care Act of 2010, Title V, defines health literacy as the degree to which an individual has the capacity to obtain, communicate, process and understand basic health information and services to make appropriate health decisions” ("What is health literacy?", 2016, para. 2). Several articles in this review cited health literacy as a barrier to patient education and self‐efficacy (Devan et al., 2018; Mackey et al., 2016; Rivera, 2017; Win et al., 2016).

Lack of access to health care was a major contributor to patient self‐efficacy. Three areas are found to be a barrier to access: (a) gaining access to a healthcare system, (b) having access to the location of specialty services needed and (c) access to a provider who one can easily communicate with and trust (“Access to Health,” 2019). The most prominent barrier related to chronic disease noted in the literature is accessed due to location (Abroms, 2019; Kennedy et al., 2017; Koch et al., 2015; Roncoroni et al., 2019; Wilson et al., 2018).

Further, patient support systems played a role in self‐efficacy. Koch et al. (2015) described support systems as being formal—health organization groups—and informal—peer groups and family. Several reviewed articles identified a lack of support, either formal or informal, being a barrier to self‐efficacy and positive patient outcomes (Cameron et al., 2018; Fors et al., 2018; Henselmans et al., 2014; Koch et al., 2015; Stellefson et al., 2017; Willis, 2016).

Research question two sought to identify what non‐traditional strategies and programmes can be implemented by healthcare leaders to promote self‐efficacy in patients with chronic disease. Five themes were identified: (a) self‐management programmes, (b) telehealth, (c) mobile applications, (d) gaming, and (e) social media. The first and a popular and proven tool in the literature for increasing self‐efficacy in chronic disease management is self‐management programmes (Anekwe & Rahkovsky, 2018; Bandura, 2005; Cameron et al., 2018; Wilson et al., 2018). Lin et al. (2017) describe this as patients performing daily care functions related to their disease processes. Although this is proven to increase self‐efficacy, there are many different strategies for self‐management plan implementation beyond face‐to‐face delivery and research found them to be underused.

Telehealth or telemedicine can be described as health information transmitted via technology to support and promote health care from a distance. ("Telemedicine and Telehealth," 2017). Interventions via telephone and video were prominent in this review (Cameron et al., 2018; Cottrell et al., 2017; Fors et al., 2018; Kennedy et al., 2017). In addition, a small number of mobile applications and gaming were identified as potential interventions to promote self‐efficacy. Two articles were found on mobile applications and one on gaming (Baron et al., 2016; Hardinge et al., 2015; Hickman et al., 2015). Although limited, findings were positive and further research is recommended.

The Internet has made a substantial impact on information exchange, and Americans have made social media platforms a part of their everyday life (Abroms, 2019). Recent survey findings identified that 42% of adults, 65 and older, now own smartphones and 67% use the Internet (Anderson & Perrin, 2017). In addition to increased access, there is also a noted increase in social media use. Facebook being the most used: 42% of adults, 65 and older, now use Facebook (Greenwood, Perrin, & Duggan, 2016). Only two studies addressed social media (van Berkel et al., 2015; Wilson et al., 2018). However, the increased use of social media and Internet access and study findings support the need to explore social media as a platform for sharing information.

## 3 DISCUSSION

The aim of this review was to investigate self‐efficacy in patients with chronic disease and answer the following research questions: (a) What are barriers to self‐efficacy in patients experiencing chronic disease? and (b) What non‐traditional strategies and programmes can be implemented by health care leaders to promote self‐efficacy in patients with chronic disease? Findings to be discussed are three significant barriers to self‐efficacy and five strategies to enhance traditional education and boost self‐efficacy. Although some strategies had limited literature available, overall, the findings highlight potential interventions healthcare leaders can implement to increase adherence to treatment regimens and decrease complications.

### 3.1 Barriers to patient self‐efficacy

Simply shifting to patient‐centred care and placing the patient at the centre to have an active role in their ongoing health care is not a one‐size‐fits‐all approach (Fors et al., 2018). When deriving strategies to improve self‐efficacy, there are many angles to consider. Not every patient will be equipped with the same level of knowledge and understanding about the disease process; therefore, they may not believe in successfully managing their care. Others may have a high level of understanding and high self‐efficacy, believing that they can manage their care; however, managing chronic disease processes can prove exhaustive and motivation can decrease over time (Devan et al., 2018). In addition, the complexities of chronic disease often require too much education for the short period of allotted time with the practitioner (Win et al., 2016). Further, new information overwhelms the patient, making crucial the timing of education (Win et al., 2016) and targeting the specific beliefs and needs of each patient in developing a plan (Fors et al., 2018).

Goals for patients with chronic diseases are to avoid complication, prevent deterioration and maintain function. Several factors such as attitude, knowledge and skills, and support and beliefs can influence one's health behaviours (Wu et al., 2016). Devan et al. (2018) found that many factors such as personal, psychological and treatment regimens can be facilitators and barriers to ongoing self‐management. And additionally, Roncoroni et al. (2019) found that self‐efficacy enhances engagement in health‐promoting activities and adherence to treatment regimens and that finding ways to boost self‐efficacy should be a priority.

### 3.2 Health literacy

According to the U.S. Department of Health and Human Services (2018), communication is complex. Although passive educational support, such as providing literature, can improve self‐efficacy and outcomes, it is not appropriate when health literacy is a factor (Devan et al., 2018). Often patients are overwhelmed with a new diagnosis and new information and not understanding this information can compound the patient's stress. This reinforces that it is pertinent for providers to understand the needs of the patient individually and providers should probe patients thoroughly to determine the appropriate interventions and improve self‐efficacy (Cutler et al., 2018).

The impact of health literacy on patient self‐management was explored by Mackey et al. (2016). It was identified that self‐managing chronic diseases requires knowledge and skill and can be acquired through different modalities of education and resource support. Results were conflicting: the findings of some of the disease categories reported higher health literacy, resulting in higher self‐efficacy, while some reports found no association. Overall, the findings support an association between health literacy and self‐management skills and recommend identifying patients with low literacy and providing appropriate interventions to improve self‐efficacy.

### 3.3 Access

Lack of access and geographical isolation restricts timely care of patients, and furthermore, rural residents have lower rates of treatment adherence and higher rates of mortality than their urban counterparts (Cottrell et al., 2017). In addition, impoverished areas were cited for having a higher risk of developing chronic health conditions than more advantaged areas. Lower‐income adults have more stress; they have fewer opportunities to engage in health promotions and limited access to healthy foods and safe areas to exercise (Horrell et al., 2017). Roncoroni et al. (2019) concluded that boosting self‐efficacy can increase adherence to treatment regimens and, therefore, is necessary to find ways to close the access gap. Kennedy et al. (2017) concluded that the self‐efficacy was improved and behaviour changes noted after a self‐management programme was delivered using telemedicine and could be the solution to reaching rural patients.

### 3.4 Support

Providing necessary interventions that improve outcomes can be difficult. Social support, Cameron et al. (2018) argue, is a key to effectiveness in self‐management programmes for patients with chronic disease. In addition, Willis (2016) posits that social connectedness and support produce greater self‐efficacy. Fortin et al. (2019) found that interventions are limited in some patients when the family is opposed to change or do not support the patient. According to Willis (2016), a person's self‐efficacy can directly influence behaviour change and encouragement or discouragement received from one's social support system has an impact on this. Insight into a patient's perceived difficulties can be valuable for a clinician to enhance their interaction with patients and improve efficacy.

### 3.5 Limitations

This review has many limitations that may limit the generalizability of this review. A limitation to this review could be the studies and reviews were used from many countries with the knowledge that the lived experience of patients with chronic disease remains independent of geographical locations. However, it is noted that some countries may not have the same access to health care nor practice the same standard of care for some disease processes and therefore may affect self‐efficacy differently. Further, it is noted that traditional education that starts with primary care may not be the standard in all areas.

In addition, a limitation could be the small number of research articles and reviews available that focus on strategies beyond traditional face‐to‐face education. Although most found increase in self‐efficacy, the studies were small and the reviews were limited due to the small number of studies conducted to date, as well as, the exclusion of studies not written in the English language. Furthermore, most literature sources were qualitative or a literature review leaving the interpretation of the authors to be subjective in analysing and identifying themes to report results.

## 4 NEW STRATEGIES

### 4.1 Self‐management programmes

Self‐management programmes are not exactly new; they have been around for several years. However, the success of these interventions is mixed (Mackey et al., 2016) and the literature review finds a wide variety of self‐management programmes and many patients with chronic disease not having access. The self‐management of complex disease processes requires individuals to identify and attempt the necessary tasks to improve their quality of life (Lin et al., 2017). Self‐management programmes were not created to replace conversations between patients and healthcare providers, but instead to complement traditional strategies (Cameron et al., 2018). Cutler et al. (2018) suggest that self‐management programmes should be implemented to decrease the number of hospitalizations and further reduce the overall costs.

According to the notion of intentional strategy, patient education cannot remain generic. It should instead be provided according to the patient as an individual and developed considering his or her particular needs (Wu et al., 2016). Roncoroni et al. (2019) found self‐efficacy to improve adherence to treatment and suggested the following ways to boost self‐efficacy: (a) breaking down the target behavior into smaller components; (b) coming up with a plan including specific behavioral strategies; (c) allowing patients to make their own choices grounded in their belief practices and developmental level; (d) giving patients consistent and focused feedback (p. 114). Therefore, maintaining a patient‐centred approach requires innovative self‐management strategies that focus on better and ongoing communication across support systems (Devan et al., 2018; Hardinge et al., 2015).

Further, an identified barrier to self‐management is access. In a study by Horrell et al. (2017), the authors noted that patients living in low‐income areas are vulnerable to numerous chronic diseases and face many barriers to self‐management. The study was significant because, in a sample of 19,365 participants in a Chronic Disease Self‐Management Course, the most impoverished completed the course more frequently than other participants. This means that barriers occur prior to enrolment in programmes and strategies to reach this population have the potential to improve health outcomes in this group.

### 4.2 Telehealth

According to Hardinge et al. (2015), telehealth applications can be easily used with both patients and healthcare providers to collaborate, manage and support self‐management of health remotely. There are many low‐cost options available that can integrate with an organization's electronic systems as well as be easy to use for patients who have basic computer skills. Moreover, Cameron et al. (2018) posit that self‐management has the potential to improve outcomes, but in rural areas, there are few people with a given chronic condition and few experts that can lead programming. Therefore, telehealth can fill the gap to reach equity in healthcare services and improve outcomes for patients with long‐term illness.

Several examples of success with telehealth were found in the literature. In a study by Cameron et al. (2018), video conference was used as a companion to a literature guide to self‐management and covered many topics including mental health. Significant group efficacy and self‐efficacy were found following the programme. In another study by Fors et al. (2018), participants were provided telephone support following hospitalization with acute exacerbations of their disease. Findings noted the intervention reduced the risk of decreased self‐efficacy and decreased clinical events during the study's duration.

Cottrell et al. (2017) reviewed studies exploring the use of telerehabilitation for musculoskeletal disease and found unequivocally that most musculoskeletal conditions can be managed through telehealth mediums and spotlights the possibilities of telehealth as a tool for patients in remote areas who do not have access to health professionals and the services. In addition, a study by Kennedy et al. (2017), which converted a 1‐day arthritis workshop into a 2‐day workshop using a video conference to reach remote patients, produced the same results and increased self‐efficacy, corroborating the data supporting telehealth strategies to improve outcomes.

### 4.3 Gaming

Limited information was found in the literature that described the use of games to improve self‐efficacy. In a study by Hickman et al. (2015), it was noted that serious games for adolescents that allowed them to learn about their disease have proven to increase self‐efficacy and behaviours, but only a limited number of similar technologies concerning adults have been tried. Participants in the study were individuals affected by chronic disease and were exposed to a serious game for health called eSMART‐HD. This chronic disease intervention system allowed participants to interact with an avatar‐based healthcare professional, allowing them to practice with the opportunity to experience an interaction based on their specific disease process. The participants received real‐time feedback and educational resources to supplement the interaction. The results were mixed, with some having positive outcomes and others having no change; however, it is noted that the use of gaming in the self‐management of chronic disease could be a promising strategy.

### 4.4 Mobile applications

DiClemente, Nowara, Shelton, and Wingood (2019) posit that new technologies may be equally, or possibly more, effective than old strategies and mobile technologies via the Internet and smartphones can potentially promote behaviour change. Baron et al. (2016) studied the implementation of a mobile phone application that allowed people with diabetes to transmit data to the healthcare provider and patients received timely feedback that identified any potential issues. The findings supported the increase in self‐efficacy and noted that real‐time education through feedback improved behaviours and treatment adherence. Themes that arose were that patients were more aware of their care and were more motivated to improve because they felt that “someone was watching”—this also made them feel more secure in their abilities. The authors posited that this strategy might empower patients to manage their disease and improve self‐care.

Another option for mobile applications is an Internet‐linked software application. Hardinge et al. (2015) studied the mHealth system that allowed the patient focus group to record data and access evidence‐based educational materials. This was noted to be a more generic technology that could be integrated into existing organizational platforms and can be easily used by patients of varying computer skills. Results found patients to be highly compliant with the use of the application, using the educational materials, submitting ongoing home assessment finding such as oxygen saturation and communicating with their healthcare providers through the communication option. This approach was found to potentially identify exacerbations in the early stages; however, as with the other studies, this strategy needs more research.

### 4.5 Social media

Limited research was found about the use of online social media as a medium for improving self‐efficacy in patients with chronic disease, and Abroms (2019) noted very little peer‐reviewed literature that looked at social media's influence on health; however, with the growing population of social media use, this could be a possible platform for sharing resources and supporting patients with chronic disease. DiClemente et al. (2019) call for a shift to technology‐based interventions as stand‐alone, or to supplement face‐to‐face or small group education, arguing that creating social networks for patients can foster continued adherence to health‐protective behaviours. Social media has been found to provide a space for patients with similar health issues to provide support for one another (Abroms, 2019; van Berkel et al., 2015).

Abroms (2019) discussed concern for the use of social media noting that when patients search for sites related to their disease process, they, unfortunately, may find the highly viewed posts first, which may not be the most helpful and may provide harmful information. Further, there are no formal systems in place that monitor posts and intervene when they are of a harmful or incorrect nature (Abroms, 2019; van Berkel et al., 2015).

A study by van Berkel et al. (2015) referred to self‐efficacy as empowerment and reviewed message boards for three prominent chronic diseases. Empowerment processes were found in all threads, and the most frequently occurring were providing information, sharing personal experience and requesting information. The authors concluded that the message boards could increase self‐efficacy, particularly in the area of medications. “No health promotion technology is perfect,”(DiClemente et al., 2019, p. 5119); not every intervention and technology is right for every patient; however, as research shows, traditional education increases knowledge but without changing behaviours, while increasing self‐efficacy can not only improve health outcomes but also change behaviours. Although social media sites do come with potential pitfalls, they are a part of the modern world and everyday communication, making it essential to identify ways to use them effectively.

### 4.6 Implications for education, research and practice

Healthcare providers must be aware of the impact of assessment for self‐efficacy in patients affected by chronic diseases. Chronic disease prevention and self‐management education still has a significant place in primary care and continues to be the starting point for patients to manage their own disease processes and increase positive outcomes (Fortin et al., 2019). The need for quality patient–provider interactions and identifying the specific needs of patients is imperative (Hickman et al., 2015).

Aging populations and increased chronic health conditions result in increased cost from readmissions and healthcare organizations continuously search for strategies to decrease costs. Self‐efficacy has proven to increase self‐management (Cutler et al., 2018). Improving self‐efficacy and self‐management programmes require healthcare organizations to break down barriers such as health literacy, access and support. The focus must be on the assessment of specific patient abilities and the need to develop a plan that patients can successfully follow to manage their disease (Fors et al., 2018).

The Internet is quickly becoming accessible to elderly patients and should be a potential strategy for filling the gap in reaching patients to implement self‐management as well as to continue to support patients and improve self‐efficacy. Although new technologies are still in their infancy and research is limited, the results found were overall positive and their potential impact should be evaluated. Further research is needed to validate the findings and guide healthcare leaders in identifying the most successful strategies.

## 5 CONCLUSION

Self‐efficacy for patients with chronic conditions has been shown to improve with interventions that support self‐management; however, it cannot be successful unless strategies are implemented to break down barriers and sustain behaviours. Bandura (2019) argues that there is limited value in attempts to change behaviour if they lack the resources and support to be successful. New programmes that help to sustain self‐efficacy must be explored by organizations to improve outcomes and decrease hospitalizations and overall costs (Cutler et al., 2018).

The integration of education for chronic disease management into primary care is essential and does increase outcomes for patients (Fortin et al., 2019). However, it is merely found to be a starting point. Patients are increasingly expected to be autonomous in the management of chronic diseases. This can prove overwhelming, with patients following complex regimens and taking directions from many specialty providers related to their disease (Bratzke, 2015). Patient‐centred care cannot be a one‐size‐fits‐all approach and organizations need to actively promote self‐management through strategies that improve and sustain self‐efficacy. New technology has been proven to enhance traditional patient education and reach more patients than ever before. Boosting self‐efficacy could potentially be a way to increase treatment adherence and outcomes and decrease cost.

## CONFLICT OF INTEREST

There is no conflict of interest to declare.

## AUTHOR CONTRIBUTION

There are no additional authors.
